# The influence of reduced graphene oxide on stem cells: a perspective in peripheral nerve regeneration

**DOI:** 10.1093/rb/rbab032

**Published:** 2021-06-25

**Authors:** Xiangyun Yao, Zhiwen Yan, Xu Wang, Huiquan Jiang, Yun Qian, Cunyi Fan

**Affiliations:** 1 Department of Orthopedics, Shanghai Jiao Tong University Affiliated Sixth People's Hospital, 600 Yishan Road, Shanghai 200233, China; 2 Shanghai Engineering Research Center for Orthopaedic Material Innovation and Tissue Regeneration, 600 Yishan Road, Shanghai 200233, China; 3 Youth Science and Technology Innovation Studio of Shanghai Jiao Tong University School of Medicine, 600 Yishan Road, Shanghai 200233, China; 4 College of Fisheries and Life Science, Shanghai Ocean University, 999 Metro loop Road Shanghai, China

**Keywords:** reduced graphene oxide, nerve regeneration, tissue engineering, biomaterials, stem cell

## Abstract

Graphene and its derivatives are fascinating materials for their extraordinary electrochemical and mechanical properties. In recent decades, many researchers explored their applications in tissue engineering and regenerative medicine. Reduced graphene oxide (rGO) possesses remarkable structural and functional resemblance to graphene, although some residual oxygen-containing groups and defects exist in the structure. Such structure holds great potential since the remnant-oxygenated groups can further be functionalized or modified. Moreover, oxygen-containing groups can improve the dispersion of rGO in organic or aqueous media. Therefore, it is preferable to utilize rGO in the production of composite materials. The rGO composite scaffolds provide favorable extracellular microenvironment and affect the cellular behavior of cultured cells in the peripheral nerve regeneration. On the one hand, rGO impacts on Schwann cells and neurons which are major components of peripheral nerves. On the other hand, rGO-incorporated composite scaffolds promote the neurogenic differentiation of several stem cells, including embryonic stem cells, mesenchymal stem cells, adipose-derived stem cells and neural stem cells. This review will briefly introduce the production and major properties of rGO, and its potential in modulating the cellular behaviors of specific stem cells. Finally, we present its emerging roles in the production of composite scaffolds for nerve tissue engineering.

## Introduction

A lot of innovations are revolving around the synthesis and applications of graphene in tissue engineering nowadays. Graphene possesses superior electrical conductivity, mechanical strength and biocompatibility [1]. These attributes endow graphene with great potential in tissue engineering and regenerative medicine. The reduced graphene oxide (rGO) is one of the graphene derivatives with great structural and functional resemblance to graphene. Besides, the residual functional groups on rGO also give it the edge over pristine graphene to accept tailored modification and functionalization. To date, many researches shed interesting light on the biomedical application of rGO, including drug delivery system, gene therapy, diagnostic contrast substance, regenerative medicine and tissue engineering [2, 3].

Nerve tissue has limited regenerative capacity after injuries and the functional restoration of injured nerves is hard to achieve. Although various treatments can repair the structural damage in peripheral nerves, the repair of long-gap peripheral nerve injuries remains a challenge. An optimal reconstruction of injured nerves necessitates early intervention [4]. Nerve tissue engineering employs biocompatible and biodegradable scaffolds to guide nerve regeneration in an appropriate direction and offer necessary nutrients, signaling molecules and oxygen [5]. However, autograft transplantation of peripheral nerve generates better clinical outcome than the transplantation of synthetic nerve conduits and is the current standard-of-care treatment for large peripheral nerve defects [6].

Schwann cells (SCs) are known for their capacity to guide the axonal regrowth and remyelination. Over the past years, SC-seeded nerve scaffolds propelled the development of peripheral nerve regeneration greatly. However, the sources of SCs are limited due to their low division rate and difficult isolation techniques [7]. Stem cells are characterized with self-renewability and can differentiate along neural lineages. In this regard, stem cells-derived SCs provide unlimited source of functional SCs for treating PNIs.

Biomaterial scaffolds facilitate the adhesion, proliferation and oriented differentiation of stem cells [8−10]. Recent advances in stem cell researches have geared the nerve tissue engineering toward the synergistic application of biomaterials and stem cells. Traditional theories considered that biochemical supplements (i.e. growth factors) dictate the fate of stem cells, while scaffold substrates simply offer platforms for these bioactive factors. However, recent studies prove that biophysical properties of scaffolds play important roles in the decision of stem cell fates [11]. A well-designed porous structure endows scaffolds with enough surface area for human mesenchymal stem cell (hMSC) adhesion and elongation [12]. The mechanical properties of scaffolds control the differentiation direction of hMSCs through the modulation of cytoskeleton dynamics [13]. Lee *et al.* demonstrated that hMSCs cultured on fibronectin and collagen tended to differentiate along the adipogenic and neurogenic lineage, respectively [14]. Significantly, the electroconductivity of materials promotes stem cell differentiation toward electro-active lineages and induces neural lineage commitment [15]. RGO is versatile biomaterial with excellent electroconductivity. Although the detailed regulatory effect of rGO in stem cell behavior remains unknown, rGO is considered as an ideal candidate in the development of stem cell-based therapy for peripheral nerve repair [16−18].

This review comprehensively collects the experiments on the interaction of rGO and several specific stem cells, including embryonic stem cells (ESCs), MSCs, adipose-derived stem cells (ADSCs) and neural stem cells (NSCs). These stem cells can further differentiate into neural cells and take part in the peripheral nerve regeneration. The synergistic effects of rGO and stem cell transplantation exhibit favorable influence on the peripheral nerve repair.

## The production and properties of rGO

Graphene consists of a single sheet of densely packed, hexagonal structured carbon atoms [19]. Such single-layer graphene possesses excellent electrical, mechanical and thermal properties and has prospective potential in biomedical application [20, 21]. There are several ways to obtain graphene directly from graphite, including chemical or mechanical exfoliation, pyrolysis, chemical vapor deposition and chemical synthesis [22]. However, pristine graphene consists of mere carbon−carbon bonds and stringently arranged aromatic structure. This stable, non-functionalized, hydrophobic structure prevents graphene from absorbing foreign atoms, receiving modification and being dispersed into other materials [23]. Therefore, a more flexible and biocompatible substitute is desired.

Surface oxidization is an important engineering method to enhance the biocompatibility of biomaterials. Natural material combined with oxide surface could increase the bioactivity and biological events at the tissue−material interface [24]. The oxygen etching treatment also controls cell adhesion and enhance the biocompatibility of inert materials. Babaei revealed that the concentration of carboxyl groups controlled the adhesion of U937 and NB4 cell lines to the surface of inert polymers [25]. The rGO is a partially oxidized derivative of graphene and shows great structural and functional resemblance to graphene. The remnant-oxygenated groups can introduce new specific functional groups onto rGO surface and thus rGO is modified either by chemical or electrochemical functionalization [26].

**Figure 1.**
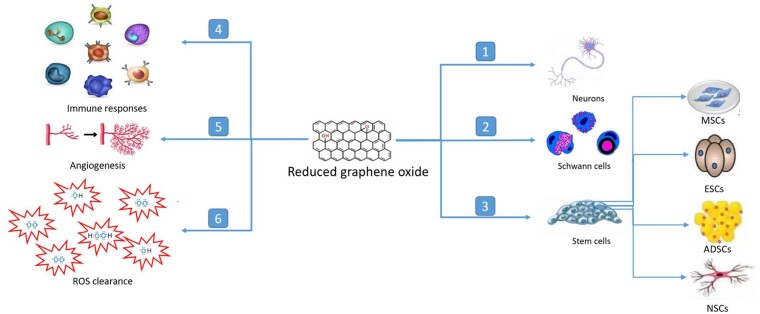
RGO regulates the cellular behaviors of neurons, SCs and stem cells in the nerve regeneration (presented in 1, 2 and 3). in the reconstruction of extracellular microenvironment, rGO stabilizes the neo-vascularization, immune response and ROS clearance in the regenerating nerves (presented in 4, 5 and 6)

The transition from graphite to rGO involves two intermediates, graphite oxide and graphene oxide (GO) [27−29]. Electroconductivity and mechanical strength are significantly enhanced through this process [30]. The production of rGO requires the removal of oxygen-containing groups from GO and the recovery of conjugated structure. There are many approaches to remove phenol hydroxyl, epoxide and carboxylic functional groups from GO: heating GO to 1000°C, exposing GO to UV light and treating GO with reducing agents. Nevertheless, the chemical method for GO reduction usually exploits toxic reductants and these chemicals can do great harm to living bodies. Therefore, it is desirable to exploit non-toxic and environmentally friendly reducing agents (e.g. dextran and l-ascorbic acid) to produce rGO [31−35]. A schematic diagram for the production process of rGO is shown in Fig. 1.

Graphene and its derivatives exhibit superior properties over traditional materials in terms of mechanical strength and electrical conductivity [36−38]. As a promising material in tissue engineering, rGO has the following desirable properties: electrical conductivity, biocompatibility, biodegradability, antimicrobial activity and pro-angiogenic property.

Electroconductive biomaterials can carry electronic currents which is considered beneficial to the regeneration of electroactive tissue [39−42]. Natural biopolymers (e.g. collagen) simulate the properties of extracellular matrix and provide ideal extracellular microenvironment. However, these biomimetic materials cannot be used alone in nerve tissue engineering due to their poor electroconductivity [43]. The strong carbon−carbon bonds in rGO allow free electrons to move between the atoms which provide rGO with excellent electrical properties. The addition of rGO into these biopolymers could markedly enhance the conductivity of scaffolds by several orders of magnitude [44, 45].

Carbon-based materials are inherently biocompatible materials and offer safe and green platforms for cell culture studies [46]. Kenry and Lim described the outstanding biocompatibility of graphene as a layered 2D nanomaterial [47]. More importantly, the biocompatibility of rGO could be enhanced through surface modification. The oxygen-containing groups in rGO structure provide rGO the opportunity to be modified [48]. For instance, dopamine could polymerize and attach to rGO in the reduction of GO. The anchored polydopamine consequently offers new binding sites for other bioactive molecules which increased the biocompatibility of rGO dramatically [49]. The toxicity of rGO composite scaffolds mainly stems from the chemical reductants left from the reduction process. Li et al. discovered that the treatment of only 1 μg/ml hydrazine-reduced rGO can destruct the cell viability in hMSCs significantly [50]. Wu et al. demonstrated that rGO reduced by sodium sulfide (Na_2_S) is more toxic to bone marrow-derived macrophages and J774A.1 cell line than GO [51].

Many studies have assessed the biodegradation of graphene and its derivatives. Enzymatic catalysis is essential to the biological degradation of carbon-based materials [52]. For instance, oxidative enzymes (i.e. myeloperoxidase) secreted from immune cells could successfully degrade different graphene-based materials [53]. However, the functionalized rGO cannot have direct contacts and interaction with enzymes due to the functional groups on its surface [50]. In this respect, the improvement of biocompatibility of graphene-based materials might attenuate the biodegradability of rGO. Besides, the degradation products of rGO include rGO fragments, carbon dioxide and polycyclic aromatic hydrocarbons, which are potentially carcinogenic and incompatible *in vivo* [54]. In fact, it is hitherto unidentified about the degradation of rGO in the living body.

Graphene-based materials have strong cytotoxicity to bacteria. GO exhibits the highest antibacterial activity, sequentially followed by rGO, while graphite has the lowest antibacterial activity [55]. The antibacterial effects of graphene-based materials are possibly attributed to the membrane stress and oxidative stress [55]. Graphene-based materials could damage the bacterial cellular membrane and oxidize cellular structure, leading to bacterial cellular death [56, 57].

RGO can also induce reactive oxygen species (ROS) and reactive nitrogen species thus modulating angiogenesis [58, 59]. Highly dosed rGO induces excessive intracellular ROS which leads to the cell death of endothelial cells and generates anti-angiogenic outcomes. On the contrary, rGO at lower concentration enhances the migration of endothelial cells and indicates pro-angiogenic properties [60]. RGO activates Akt phosphorylation to enhance the phosphorylation level of nitric oxide synthase (NOS). NOS mediates the production of nitric oxide (NO) which promotes physiological and pathological angiogenesis [61, 62]. Overall, the characteristics and properties of rGO makes it an appropriate and accessible material for tissue engineering and regenerative medicine.

## The interaction between rGO and stem cells in peripheral nerve regeneration

The treatment of peripheral nerve injury remains a challenge because few satisfactory therapies can meet the ever-stringent requirements of regeneration in long nerve defects. A permissive regeneration environment is the key to initiating the process of peripheral nerve regeneration. The peculiar properties of rGO can balance the injury-induced microenvironment through various intracellular and extracellular changes. Many studies have reported these changes, but the underlying mechanisms remain unclear. In this context, this review proposes a possible mechanism for rGO composite scaffolds to induce regeneration: rGO generates various cellular responses at cell−material interface, and at the same time, rebuilds the extracellular microenvironment in terms of angiogenesis, anti-inflammation and anti-oxidative stress.

Neo-vascularization provides nutritional support to the regenerated peripheral nerves. RGO impregnated-GelMA hydrogels enhanced micro-vessel formation and promoted the viability, proliferation and migration of endothelial cells [63]. RGO loaded isabgol nanocomposite scaffolds exhibited excellent angiogenic properties in which accelerated blood vessel formation and increased vascularization density were observed histologically [64]. Macrophages play a key role in orchestrating the injury-induced peripheral nerve regeneration, and rGO possesses the ability to modulate macrophage activity [64]. Serrano et al. found that rGO promoted the accumation of pro-inflammatory M1 macrophages at the early phase of repair, followed by the shift to anti-inflammatory M2 phenotype [65]. PNI disrupts the mitochondrial function and renders excessive exposure of ROS. Although mildly oxidative environment may promote the angiogenesis, excessive ROS exposure impairs neurite outgrowth and SC remyelination [66]. RGO was found to scavenge intracellular ROS efficiently at a specific range of dose [67]. Kang et al. verified that rGO could react with cellular ROS in neuronal cells and then was transformed into oxidized rGO [68].

The initiation of nerve regeneration also includes the restoration of bioelectricity. Appropriate electrical currents are beneficial to the regeneration of electroactive tissues. Single-layered graphene scaffolds significantly increased neuronal membrane ion currents and neuronal excitabilities [69]. Besides, rGO modulated the neurotransmission of neuronal cells through direct contact with actin dynamics, the structural basis for membrane activities (e.g. neurotransmitter release) [68]. Furthermore, rGO scaffolds not only facilitate the reconstruction of electrophysiological function, but also assist in the neuronal regrowth [70]. Lovat et al. clarified that electroconductive carbon substrate boosted neuronal electrical signaling and caused the redistribution of charges along the surface of the membrane [71]. Electrical stimulation could also upregulate the expression of pro-neurogenic proteins (e.g. brain-derived neurotropic factor, tyrosine kinase B, α1-tubulin and growth-associated protein 43) in neurons [72]. Mitogen-activated protein kinases (MAPKs) was able to transduce electrical stimulation into intracellular signaling pathways [73]. Additionally, c-Jun N-terminal kinases (JNKs) is known to regulate inflammatory responses, axonal regeneration and myelination. Zhao et al. clarified that electrical stimulation at electroconductive scaffolds led to the activation of MAPKs and the attenuation of JNKs in the regenerated peripheral nerves [74].

SC is the main supporting cell for peripheral nerve regeneration. Recently, Fang et al. discovered that SCs cultured on rGO upregulated the expression of epithelial-mesenchymal transition-related genes, an indication of reprogramming toward invasive stem cell-like cells. In their study, rGO increased the expression of SOX-2, which was usually activated in dedifferentiated SCs in the peripheral nerve repair [75]. Our previous work also discovered the increased gene expression of myelin protein and neurotrophic factors in rGO-cultured SCs [76]. An enhanced gene expression of nerve growth factor (NGF), peripheral myelin protein 22 (PMP22) and early growth response 2 (Krox20) were detected by real-time PCR and western blot. These genes are frequently associated with myelination. However, the expression of neuronal cellular adhesion molecules, an indicator of immature SCs, significantly decreased on the rGO composite scaffolds compared with that on the control. Based on the facts mentioned above, rGO provides a supportive milieu for nerve regeneration with synergistic effects of angiogenesis, inflammatory modulation, metabolism stabilization and bioelectricity reconstruction (Fig. 1).

Stem cell-based therapies are at the forefront of healing the nerve tissue. Nerve tissue regeneration can be achieved by the differentiation of specific stem cells, including ESCs, MSCs, ADSCs and NSC. Stem cells characterize self-renewal ability and multiple differentiation potential. Biomaterial scaffolds facilitate the various cellular behaviors of stem cells as cell culture platforms. RGO composite scaffolds are progressively successful in the regulation of stem cell fate, such as cell growth, differentiation and proliferation [77]. Given the background that rGO provides pro-neurogenic milieu, the combination of stem cells and rGO generates synergistic effect in the promotion of peripheral nerve regeneration. Herein, we comprehensively review the interaction between rGO and specific stem cells and list the pro-neurogenic effects of rGO on stem cell fates in Table 1.

**Table 1.** The influences of rGO on four different stem cells

Cell type	Scaffold	Effect	Related mechanisms	References
ESC	Porous rGO substrate	Maintenance of pluripotency; promotion of adhesion and proliferation	Activation of the E-cadherin/Wnt signaling pathway; activation of integrin signaling pathway, with decreased expression of Vinculin and MEK1	[78−80]
MSC	3D rGO-PADM hybrid scaffolds; electrical stimulation assisted rGO-PEDOT, hybrid scaffolds	Promotion of neuronal differentiation, adhesion and proliferation; neurite sprouting and outgrowth; acceleration of the osteogenic differentiation	Enhancement of Nestin, β-tubulin III and MAP2 expression; activation of the mechanosensitive integrin-FAK axis	[81−85]
ADSC	3D alginate/rGO hybrid scaffold; 3D cellulose/rGO hybrid scaffold; rGO mat	Promotion of proliferation, neurogenic differentiation, osteogenic differentiation and mineralization; increased synthesis of NGF	Enhancement of intracellular calcium concentration; secretion of exosomes	[86−89]
NSC	Nanostructured rGO-based microfibers; silk nanofibers/rGO hybrid scaffold	Promotion of adhesion, proliferation and differentiation into both glial cells and neurons; formation of a strong neural network	Activation of the integrin-mediated interactions between NSCs and scaffolds; enhancement of β3-tubulin expression	[90−92]

### Embryonic stem cells

ESCs are pluripotent stem cells derived from the inner cell mass of blastocysts and differentiate into all types of somatic cells. The great versatility equips ESCs with the ability to regenerate or repair injured tissue and organs. ESCs can be exploited to obtain any desired cells from three cellular layers of endoderm, mesoderm and ectoderm [93]. In this context, current researches of ESCs mainly revolve around its large-scale expansion and the pluripotency maintenance. However, ESCs have a tendency to differentiate spontaneously under *in vitro* culture conditions and gradually lose their pluripotency in this process [94]. Porous rGO composite scaffolds can maintain the pluripotency of ESCs for a long time without the destruction of ESC self-renewal ability and multi-lineage differentiation. The activation of E-cadherin/Wnt signaling pathway conducted by rGO plays a pivotal role in this process [78]. E-cadherin mediates the calcium-dependent cell−cell contact and binds to β-catenin at the same time [95, 96]. β-catenin then interacts with specific cytokines and regulates the expression of downstream Wnt targeting genes [97, 98]. GO maintained the stemness and self-renewal ability of ESCs through the integrin signaling pathway which down-regulated Vinculin and decreased the expression of MEK1 [79]. A dose-dependent promotion of neural differentiation was discovered in ESCs by adding a dose of 1, 10, 20, 50 and 100 µg/ml GO [80]. GO effectively enhanced the dopamine neuron differentiation proved by the results of immunocytochemistry and real-time PCR. Nonetheless, the ethical issues of embryo manipulation bring ESCs-related scientific researches a lot of controversies.

### Mesenchymal stem cells

MSCs are adult stem cells isolated from bone marrow, adipose tissue, umbilical cord tissue or amniotic fluid. MSCs differentiate into mesenchymal originated somatic cells (e.g. osteoblasts, chondrocytes and myocytes) and therefore take part in musculoskeletal tissue regeneration. In the recent years, new discoveries reported MSCs could also secrete neurotrophic factors and differentiate into non-mesenchymal originated nerve cells [99−101]. Hence, MSCs hold great promise as a treatment option for nerve injuries. RGO-related composite scaffolds can mimic the extracellular matrices that orient the cellular behavior of MSCs. MSCs seeded on rGO/PADM (porcine acellular dermal matrix) expressed stronger neural markers, including Nestin, β-Tubulin III (Tuj1) and microtubule-associated protein-2 (MAP2), relative to MSCs on PADM scaffolds [81]. RGO-PEDOT (3,4-ethylenedioxythiophene) hybrid scaffolds can increase the electrical conductivity of cell culture medium and facilitate the neural differentiation of MSCs [82]. Nayak et al. found out that the combination of graphene-coated scaffolds and osteogenic medium generated synergized effects and remarkably accelerated the osteogenic differentiation of MSCs. The function of rGO in this process was comparable to the pro-osteogenic effect of bone morphogenetic protein-2 (BMP-2) and rGO was not detrimental to the viability of MSCs [83]. MSCs cultured on stiffer medium often exhibit greater potential of osteogenic differentiation [84]. Xie et al. transplanted MSCs loaded graphene scaffolds into the subcutaneous tissue of severe combined immunodeficiency mice. Bony-like structures appeared on the scaffold surface and exhibited positive expression of Runt-related transcription 2 (RUNX2) and osteopontin (OPN) (both are markers for osteogenic differentiation) [85]. They further discovered that graphene triggered the activation of ‘integrin-FAK’ (focal adhesion kinase) axis and this mechano-sensitive axis promoted the osteogenesis of MSCs without the addition of any exogenous chemical inducers (e.g. BMP-2). Based on these observations, the scaffold architecture (e.g. stiffness, porosity and surface area), the material attributes, the biochemical factors (e.g. neurotrophic factors and osteogenic growth factors) and the cell-scaffold interactions are all influential factors on the MSC lineage commitment. Electrical stimulation and electrically conductive cell culture medium promote neural differentiation of MSCs. Therefore, the addition of electrically conductive rGO in composite scaffolds might orient or facilitate the neural differentiation of MSCs on neurogenic medium.

### Adipose-derived stem cells

ADSCs are considered as abundant stem cell source and differentiate into neuron-like cells after the peripheral nerve injuries [102, 103]. Some literatures reported that ADSCs could grow into neurospheres and then transform into ‘SC-like cells’ *in vitro* [104, 105]. Besides, ADSCs secrete exosomes to reduce the expression of apoptosis-related mRNA and promote proliferation of SCs. It is crucial to find an effective way to accelerate the differentiation of ADSCs due to the inefficiency in the process of ADSC differentiation. Graphene and its derivatives are considered as excellent candidates to accelerate this process [106]. Graphene-based material presented more efficiency than drugs in the promotion of ADSC neurogenic differentiation [86]. ADSCs could differentiate into functional SCs under the assistance of a bunch of glial growth factors. These SC-like cells express glial markers (e.g. GFAP) and facilitate the neurite outgrowth of motor neuron-like cells [107]. Researches further proved that SC-like cells also secrete exosomes similar to that of SCs. The mRNAs and miRNAs in the exosomes of SC-like cells can promote nerve regeneration [108]. RGO opens the calcium channels on the cells to enhance the cellular electrical interfacing and intracellular calcium concentration [87, 88]. It is reported that rGO promotes the synthesis of NGF and neurogenic differentiation through the enhancement of intracellular calcium [86]. The elevated intracellular calcium activates the calmodulin kinase and consequently affects the protein kinase expression and microRNA translation. Apart from nerve regeneration, rGO could also modulate the cellular behavior of ADSCs in bone regeneration. RGO coated, biopolymer-based hybrid scaffolds support the proliferation, osteogenic differentiation and mineralization of ADSCs [89]. ADSCs are multipotent stem cells and differentiate into specific cell lineages. Therefore, it is a great challenge to guide the differentiation of mesoderm-originated ADSCs into ectoderm-originated neural cells. Current researches could only induce ADSCs into neuron-like or SC-like cells under the condition of biomaterials, specific growth factors and electrical stimulation. However, it is expectable to fabricate rGO composite scaffolds that exhibit better performance in neurgenic differentiation guidance.

### Neural stem cells

Human NSCs are distributed in the adult central nervous systems, such as cerebral cortex, and are relatively hard to attain. Some researches investigate the cellular behaviors of NSCs in anticipation to dig out their regenerative potential. Different from the above-mentioned stem cells, NSCs only differentiate into neurons, glial cells and SC-like cells [109]. These SC-like cells could function as normal SCs in the promotion of nerve regeneration and neurite outgrowth [110]. Transplanted NSCs also secrete multiple neurotrophic factors to facilitate the repair of peripheral nerve injury [111]. Neural guide scaffolds support the proliferation, adhesion and differentiation of NSCs [112]. NSCs seeded on the rGO-based microfibers can differentiate into both glial cells and neurons at the same time [90]. Likewise, graphene-based nanotubes facilitate the neuronal differentiation and synapse formation through the activation of integrin-mediated interactions between NSCs and nanotubes [91]. The rGO-based hybrid scaffolds could enhance the expression of β3-tubulin, an early marker of the oriented neuronal differentiation. Neurite outgrowth of SH-SY5Y cells and the neural network formation were also observed in the same study [92]. Similarly, rGO microfibers induced the adhesion, proliferation and differentiation of NSCs and formed a strong neural network around the microfiber [90]. NSCs can spontaneously differentiate into neural lineage cells without the addition of specific growth factors or chemical inducers in the culture medium. Although rGO promotes the differentiation of NSCs into both neurons and glial cells, the neuronal differentiation was more frequently observed in the researches. As a member of nervous system, NSC displays unique bioelectrical properties compared with other stem cells. RGO might serve as an electrically conductive tool to guide the NSC differentiation toward neural lineage. So far, NSCs have excellently repaired many central nervous system injuries, therefore, we think highly of their future use in peripheral nerve regeneration.

## Current application and future perspectives

Graphene and its derivatives exhibit great versatility in peripheral nerve regeneration and nerve tissue engineering. Given the background that GO possesses intrinsic biocompatibility and facile functionalization, in many works, GO composite scaffolds have been used in the peripheral nerve regeneration. GO has an excellent dispersive property and solubility due to the oxygenated groups on the surface [113]. The removal of oxygenated groups from GO allows it to be reduced so that the electrical conductivity is enhanced over several orders of magnitude. However, this process also increases the hydrophobicity of the materials [114]. In this context, rGO has higher electrical conductivity but less biocompatibility compared with GO. Consequently, GO composite scaffolds have better performance in the direct cell−material contact and cell viability, while rGO composite scaffolds promote the cellular electrical activities. Particularly, neural precursor cells seeded on rGO composite membranes presented higher excitability, neural activity spikes and neural differentiation [115].

The remnant-oxygenated functional groups allow rGO to be dispersed into various matrices. In the current application of nerve composite scaffolds, there are many effective techniques to incorporate rGO into substrates. RGO can be coated onto nanofibrous substrate through the *in situ* reduction of GO to rGO. Briefly, substrates are immersed in the GO solutions to make GO-coated composite scaffolds. Then GO-coated composite scaffolds are further immersed in reducing agents (e.g. ascorbic acid) to reduce GO. Finally, the rGO-coated composite scaffolds were implanted into rat models in which 1 cm long sciatic nerve defects were created. These scaffolds exhibited similar reparative capacity to autografts [76]. RGO composite scaffolds could also be fabricated with 3D printing technology. In the fabrication of 3D printed rGO composite scaffolds, rGO powder was added with substrate pellets to produce hybrid bioink. Then the hybrid solution was subjected to the 3D printing jet to finally produce 3D culture with rGO [77]. RGO composite scaffolds could also be fabricated via electrospinning strategy. A mixture solution of certain volume of rGO and matrices was utilized in the electrospinning process, to produce hybrid nanofiber films. Afterwards, the rGO composite films were rolled into nerve guide conduits. *In vivo* results confirmed that such rGO hybrid conduits conferred a positive effect on the repair of 1 cm sciatic nerve defect with an outcome a little bit inferior to the autologous nerve transplantation [75]. In order to fabricate matrix/rGO composite membrane, one can also add matrix materials into rGO solvent to create hybrid solution. Then the well-dispersed solution was subjected to the subsequent casting and coagulation procedure to make composite membrane [115].

Over the years, rGO composite scaffolds have provided remarkable pro-neurogenic milieu which supported various activities and behaviors of cells (e.g. SC, neuron, PC12). Biomimetic scaffolds could simulate the cellular microenvironment of stem cells and provide preconcentration platforms for various growth factors or chemical inducers. The synergistic effects of rGO and polymeric biomaterials control stem cell fate through cell−material interactions. In this review, we outlined several types of stem cells applied in the field of nerve regeneration, including ESCs, iPSCs, MSCs, ADSCs and NSCs. A variety of rGO-related scaffolds were listed to exemplify the interaction between different stem cells and rGO. However, the underlying mechanism of rGO in the control of stem cell fate remains unknown. Furthermore, the *in vivo* performance of stem cells needs more studies to reveal. This brief review does not cover the full range of rGO composite scaffolds. Its destination lies in the illustration of tremendous potential of rGO. Hopefully, this review can bring more attention to the combination of rGO and stem cells as a promising strategy in the treatment of PNIs.

## Author Contributions

Y. Qian conceptualized the study. Y. Qian, X. Yao, Z. Yan, X. Wang, and H. Jiang reviewed the literature and designed the figure and table. X. Yao drafted the manuscript. Y. Qian and C. Fan revised the manuscript. All authors read and approved the final version.

## Funding

The study was sponsored by the Shanghai Sailing Program (No. 20YF1436000) and Projects of National Natural Science Foundation of China (Grant Nos 82002290 and 81830076), Municipal Hospital Newly-developing Cutting-edge Technologies Joint Research Program of Shanghai Shenkang Hospital Development Center (No. SHDC12018130), Special Fund for Research on People's Livelihood (Medical Treatment and Public Health) of Shanghai Pudong Science, Technology and Economic Commission Scientific and Technological Development Fund (No. PKJ2018-Y52), and Shanghai Pudong Health Commission Special Program for Clinical Research in the Health Industry (No. PW2018E-01). We appreciate the support from Base for Interdisciplinary Innovative Talent Training, Shanghai Jiao Tong University and Youth Science and Technology Innovation Studio of Shanghai Jiao Tong University School of Medicine.

*Conflict of interest statement*. None declared.
